# Impact of Female Gender in Inflammatory Bowel Diseases: A Narrative Review

**DOI:** 10.3390/jpm13020165

**Published:** 2023-01-17

**Authors:** Lisa Lungaro, Anna Costanzini, Francesca Manza, Marianna Barbalinardo, Denis Gentili, Matteo Guarino, Fabio Caputo, Giorgio Zoli, Roberto De Giorgio, Giacomo Caio

**Affiliations:** 1 Department of Translational Medicine, St. Anna Hospital, University of Ferrara, 44124 Ferrara, Italy; 2 National Research Council, Institute for the Study of Nanostructured Materials (CNR-ISMN), Via P. Gobetti 101, 40129 Bologna, Italy; 3 Department of Internal Medicine, SS Annunziata Hospital, Cento, University of Ferrara, 44042 Ferrara, Italy; 4 Centre for the Study and Treatment of Alcohol-Related Diseases, Department of Translational Medicine, University of Ferrara, 44121 Ferrara, Italy; 5 Mucosal Immunology and Biology Research Center, Massachusetts General Hospital-Harvard Medical School, Boston, MA 02114, USA

**Keywords:** inflammatory bowel disease, Crohn’s disease, ulcerative colitis, female sex, female gender, women, quality of life

## Abstract

Inflammatory bowel diseases show a gender bias, as reported for several other immune-mediated diseases. Female-specific differences influence disease presentation and activity, leading to a different progression between males and females. Women show a genetic predisposition to develop inflammatory bowel disease related to the X chromosome. Female hormone fluctuation influences gastrointestinal symptoms, pain perception, and the state of active disease at the time of conception could negatively affect the pregnancy. Women with inflammatory bowel disease report a worse quality of life, higher psychological distress, and reduced sexual activity than male patients. This narrative review aims to resume the current knowledge of female-related features in clinical manifestations, development, and therapy, as well as sexual and psychological implications related to inflammatory bowel disease. The final attempt is to provide gastroenterologists with a roadmap of female-specific differences, to improve patients’ diagnosis, management, and treatment.

## 1. Introduction

Inflammatory bowel diseases (IBD) are a heterogeneous group of chronic and relapsing intestinal diseases that consist of two main clinical phenotypes: Crohn’s disease (CD) and ulcerative colitis (UC) [1]. As in other immune-mediated diseases, gender affects the disease onset, course, and complications, including compliance with medical and surgical therapies [2]. Indeed, the female gender appears to be a promoting factor in CD onset, whereas it may exert a protective role in UC. Albeit the molecular pathways underlying these differences have not been unveiled yet, hormones and sex-specific differences in the immune system have been advocated to play a pivotal role in the onset and subsequent development of IBD [3].

This review describes the role and peculiar characteristics of the female sex in IBD. We will start by explaining how the female gender affects IBD epidemiology, phenotype, and activity; then, we will discuss clinical features, therapy, treatment adherence, and psychosocial aspects associated with IBD. Finally, we will illustrate how IBD affects women’s perceived quality of life (QoL). Altogether, our task is to provide gastroenterologists with a thorough update on gender-related IBD differences, including the management of female patients.

## 2. Methods

**Figure 1:**
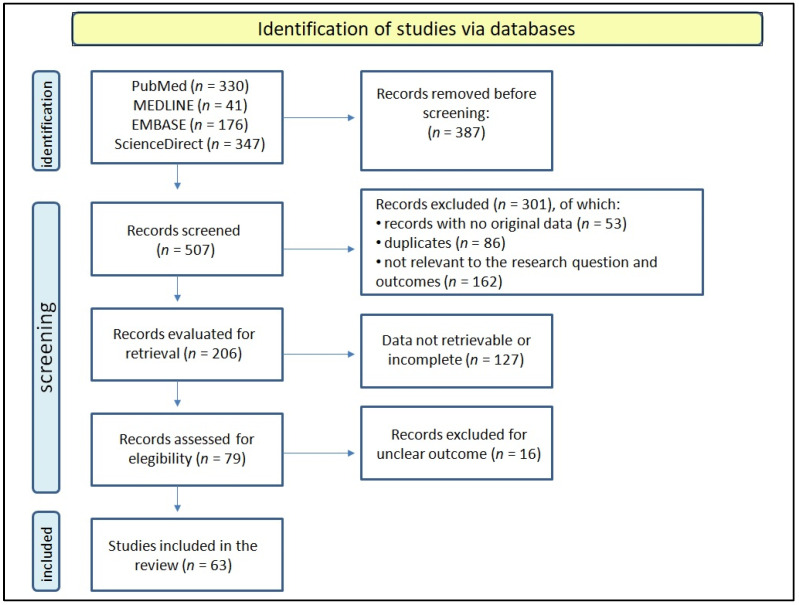
A schematic representation illustrating the process of study selection.

In this narrative review, we conducted a PubMed, EMBASE, MEDLINE, and ScienceDirect search from the inception of March 2022 to the end of August 2022 using the following search terms: “IBD” AND “female” AND “gender” OR “sex”. Literature research was carried out on the title and abstract. We also evaluated the reference list of each of the collected papers to find any other pertinent articles. The following criteria restricted the search strategy: (1) reported female-specific differences in IBD; (2) articles published in the last ten years (January 2012–August 2022); (3) material written in English; (4) full text available. The article search was carried out independently by two authors (LL and GC) who screened the titles and abstracts of the selected records. The records identified at the first step were then fully evaluated, considering the manuscript and appendices. Disagreements were resolved by consensus, and if not the case, the opinion of a third author (RDG) was considered. Non-pertinent papers or those not matching the inclusion criteria were excluded. Duplicates, papers with no original data, incomplete or with unclear outcomes were also excluded. The description of the process of study selection and the number of records found in each database are summarized in Figure 1.

## 3. Sex-Related Differences in IBD Epidemiology and Pathogenesis

IBD epidemiology is complex. The prevalence is related to the type of disease studied (e.g., CD instead of UC), the geography of the investigated area, and the age of the considered subjects. Gender-related differences have been found in the epidemiology of CD but were not so marked in UC [4]. Indeed, except for two studies observing a male preponderance over the UC epidemiology [5,6], investigations conducted both in East and West countries agreed that there was no gender-based disparity in the epidemiology of UC [7,8,9]. Shah et al., in a pooled analysis of population-based studies on 95,605 and 112,004 incident cases of CD and UC, respectively, found that age at UC onset varied with sex. Indeed, the incidence of UC is similar between males and females until age 45. After this age, females showed a 13% to 32% lower likelihood of being diagnosed with UC than males [10].

In contrast, there are sex-specific differences in CD, with women predominating in the USA and European countries [9,11,12,13,14,15], conversely to what occurs in Asian countries, where males are more prone to CD [16,17]. This evidence suggests that CD occurrence is not strictly sex-related, but other environmental factors, such as the westernization of lifestyle, could play a pivotal role in IBD pathogenesis. A recent pooled analysis of population-based studies considering 17 distinct cohorts of patients from 16 regions of Europe, North America, Australia, and New Zealand suggested that puberty in women is a trigger for CD onset, as the risk of developing the illness is 20% lower in girls than boys at age 10–14, but it markedly increases for women reaching the age of 25–29, and in particular after 35 years. A Dutch study investigating a large sample size (*n* > 5700) and two independent cohorts confirmed a low risk for very young women. The analysis revealed a male prevalence in early-onset CD (<16 years) compared to females (20% vs. 12%, *p* < 0.01) [18]. These findings support the hypothesis that female sex hormones play a role in IBD pathogenesis.

## 4. Genetics

Genetic factors are involved in IBD etiology. In familial clusters of IBD, a female preponderance has been reported (61% vs. 54% compared to sporadic IBD, respectively) [19]. The increased relative risk for females is promoted by sex-determined epigenetic factors responsible for the female-to-female transmission pattern. This aspect, referred to as “female imprinting”, was found to be particularly recurrent in familial CD (31 mothers vs. 14 father-to-child transmissions, respectively; *p* = 0.016) [19]. Moreover, several gene variants contribute to the family-specific risk of IBD. An Italian study on 203 sporadic UC patients and 391 controls correlated interleukin (IL)-10 promoter polymorphisms to a higher risk of developing UC at younger ages in females. This effect may be enhanced by estrogens that inhibit the production of anti-inflammatory cytokines, i.e., IL-10 [20]. Another study on the Pennsylvania population showed that women carrying a variant of the IL-23 receptor are protected against CD and IBD overall. Another variant of the same receptor is particularly protective against UC [21]. Intriguingly, some genetic mutations appear to be only male-specific. A study on 61 IBD patients and 101 subjects without IBD from the Lower Silesia region revealed that the *3435CT* polymorphism of the *ABCB1* gene is an IBD and CD-promoting factor in males. In contrast, the *3435TT* polymorphism of the same gene is associated with a reduced risk of IBD in men [22]. Also, compared with 139 healthy controls, a pedigree genotyping of 105 IBD subjects showed a male predominance associated with the *DLG5 R30Q* variant, confirming previous findings [23]. The same variant has been indicated as a protective factor in female pediatric CD onset [24].

The female genetic predisposition to develop IBD is also related to X chromosome abnormalities, such as Turner syndrome [25]. In IBD-affected relative pairs, X-linked susceptibility loci have been identified [26,27]. In a murine model, the loss of one allele of the Cosmc X-linked gene evoked gut dysbiosis, enhanced experimental colitis, and a spatial pattern of dysbiosis resembling IBD in males but not in female mice. One allele of this gene that encodes for a chaperone involved in the sex-specific risk of CD and UC was found to be protective against an IBD-like phenotype only in female mice [28]. These findings suggest that in females, other mechanisms regulate the enteric mucosa (broadly “glycocalyx”) and neuromuscular layer integrity. While mosaicism strengthens immunity in females vs. males, an inappropriate inactivation of some regions of the X chromosome could lead to an immune system derangement. This condition promotes a breakdown of self-tolerance [29] and favors autoantibodies [30] and the genesis of immune disorders, such as primary biliary cholangitis, autoimmune thyroid disease, Reynolds syndrome, and systemic sclerosis [30,31]. Although the complex mechanisms involving the X chromosome and autoimmune diseases have not been elucidated yet, a possible direction for further studies is represented by the naturally occurring variations in X-related genes and microRNAs taking part in the immune system arrangement. Unraveling these mechanisms may aid in a better understanding of differences between genders. Studies evaluating the incidence and prevalence of IBD by gender are summarized in Table 1.

## 5. Disease Phenotype and Complications in Females

Studies aimed to demonstrate a possible relation between gender and IBD development reveal contrasting results. In some investigations, the female gender seems to act as a protective factor, while other studies suggest the opposite [32,33,34,35]. Moreover, clinical data suggest that the IBD phenotype may show gender specificity, as males tend to manifest CD in the upper GI tract [36] or ileal region [18] compared to females. Moreover, females seem to be more protected against colorectal cancer than men [37], but not from pulmonary cancer [38].

Extraintestinal manifestations (EIM) associated with IBD appear sex-dependent and affect women more than men [18]. Females actually show a higher risk of developing eye disorders, erythema nodosum, and pyoderma gangrenosum than males. Conversely, the latter are more prone to develop primary sclerosing cholangitis and ankylosing spondylitis [36].

IBD is frequently associated with complications, such as cumulative medication use, surgery, and disease recurrence after surgery [32]. A study on 260 CD patients with a follow-up of 12 years found that IBD complications affect more men than women [33]. Moreover, a second study from the Mayo Clinic supports these findings, describing a male preponderance over major abdominal surgery, including bowel and ileocecal resection [39]. A Dutch IBD biobank study showed that the male sex was more commonly associated with small bowel and ileocecal resection [18]. However, these results were contrary to the data obtained in another study on a more extensive cohort (*n* = 1106 patients), which reported no gender-specific differences in IBD complications [32]. Osteoporosis and, in particular, osteopenia were surprisingly more common in men than women (55.9% vs. 29.6%, respectively, *n* = 174) [40]. Similar results were also found in other series, suggesting that osteopenia and osteoporosis should always be investigated in male IBD patients as they show a high risk of mineral bone abnormalities [41,42,43,44,45,46].

## 6. IBD Medical Treatment

Several pharmacological and nutritional approaches are currently available to handle IBD, but their efficacy is still under evaluation because of the novelty of some of them [47,48]. However, clinical data show that IBD management and therapy differ between males and females. Females receive fewer IBD-specific treatments than males, while major abdominal surgery is performed more frequently in men than women [18,34,39,49]. A study on 986 outpatients reported that women received less immune-suppressive treatment despite their higher disease activity [34]. Many reasons may be advocated for this finding in men: (a) higher risk of developing severe disease; (b) lower compliance to corticosteroids and/or aminosalicylates; and probably (c) lower propensity to treat women of childbearing age with immunosuppressants [34]. These possible explanations were recently tested by a Canadian study in South-West Ontario [50], examining over 1000 IBD participants. The results demonstrated that women were more commonly treated with budesonide, while men were treated with prednisone, as also confirmed by Severs et al., 2018 [18]. Moreover, the use of immunomodulators is predominant in men vs. women (86.6% vs. 78.3%; *p* = 0.008), and, nevertheless, women were more prone to experience adverse drug reactions (29.5% vs. 21.2%; *p* = 0.01) [50]. The same study also found that age is predictive of biologics treatment in women, as those over 55 less frequently receive biologics. Overall, women responded better to treatment than men [51], but they displayed lower adherence to biological treatment [52]. On the other hand, males tolerate drug therapy better than females, who reported more prominent side effects. Studies assessing IBD clinical features and management are summarized in Table 2.

## 7. IBD and Female Infertility

Infertility is described as the impossibility of conceiving after 12 months of regular, non-protected sexual intercourse [53]. Infertility rates among IBD women were reported to range from 3–14% in CD and 1.7–15% in UC, which are comparable with rates in the general population (2.5–14%) [54,55,56,57,58]. Thus, IBD females have a fertility rate comparable to the overall population, except for women with active disease or ileal pouch–anal anastomosis (IPAA), who have higher infertility rates [54]. Studies assessing serum anti-Mullerian hormone (AMH) levels as a marker of ovarian reserve and women’s fertility support these results. Data showed similar AMH levels between IBD and healthy women, indicating no differences in infertility rates due to the pathological condition. However, a retrospective case-control study measuring AMH in 50 women with CD in remission and 163 control women with physiological ovarian reserve (matched by age) concluded that hormone levels were significantly lower in ≥30-year-old women with colonic CD involvement compared to the controls [59]. Moreover, AMH levels were lower in active disease and inversely correlated with the Crohn’s Disease Activity Index, suggesting that the active disease may compromise fertility [60,61,62].

Although evidence indicated no differences in fertility rates between IBD and healthy women, patients showed half the number of children vs. healthy women, a feature known as “voluntary childlessness” [58]. This lifestyle may be explained by both mechanical and psychological grounds. From a mechanical point of view, surgical interventions greatly affect women’s anatomy, significantly reducing their ability to conceive. UC women undergoing deep pelvic dissection show a higher risk of pelvic adhesions, formation of scar tissue, post-operative dyspareunia, tubal obstruction, or alteration of the tubal-ovarian crosstalk, causing a threefold reduction in the fertility rate [63,64]. A systematic meta-analysis on the relative risk of infertility post-ileal pouch-anal anastomosis in women with UC showed that infertility increased from 15% to 48% [65]. In agreement with this result, a systematic literature search considering 22 studies reported increased infertility from 12% before restorative procto-colectomy to 26% after the intervention [63]. Moreover, a retrospective study investigating seventy-one women who had undergone procto-colectomy and ileostomy for UC and CD reported a reduction from 72% to 37% in fertility after surgery [66]. On the other hand, the psychological reluctance to conceive may depend on an altered perception of reality, which leads to an unjustified fear of the hereditary transmission of IBD, congenital abnormalities, pregnancy risks, worsening of the IBD condition during the pregnancy, and medication teratogenicity [67]. Regarding IBD heritability, available data suggest only a partial influence of the genetic components on disease onset, with high chances that the child will not develop the disease (91% if only one parent is affected and 60% if both parents are affected).

## 8. Pregnancy

Studies on IBD and pregnancy are contradictory. Some evidence suggests that conception occurring during the phase of active disease leads to a relapse of the illness in 2/3 of patients, with symptoms worsening in more than 60% of cases [68]. Moreover, the state of active disease at the time of conception could negatively affect the fetus, increasing the risk of miscarriage and reducing birth weight and pre-term birth [69]. Conversely, some studies have described a positive effect of pregnancy on IBD symptoms, especially when gestation starts during a period of disease remission. The gestation effects on IBD pathology are reported to be positive also when pregnancy starts during active disease, leading to remission in more than 70% of women with CD and more than 65% of women with UC [53,67,69]. Indeed, the intensification of symptoms during pregnancy is only transitory, and it appears during the first trimester, mainly provoked by the discontinuation of the maintenance therapy.

With regard to the worsening of the IBD condition, women conceiving during illness remission have the same chance of exacerbation as non-pregnant patients with IBD [53,67,69,70]. Pregnancy-induced positive effects are long-term, influencing the disease symptoms over a period that may last years. Available data show a reduction in the rates of stenosis and resection and annual exacerbation rates (from 0.34 to 0.18 in UC and from 0.76 to 0.12 in CD) [69,71]. The reasons underlying these findings remain elusive, but they could be associated with the positive effect of pregnancy on the immune system and the beneficial role that sex hormones exert on IBD symptoms.

Indeed, studies in animal models showed that the progressive increase of estrogen and progesterone throughout gestation decreased pro-inflammatory cytokine production and improved intestinal epithelial barrier function, reducing bacterial translocation and IBD activity at the end of pregnancy [54,72]. Despite concerns about continuing drug medication during pregnancy and breastfeeding, data indicate that, except for methotrexate, drugs used for IBD treatment are generally safe and do not increase the risk of congenital abnormalities or adverse effects on the fetus. The Toronto and ECCO consensus statements recommend continuing thiopurines, or anti-TNF alpha agent therapies during pregnancy in well-controlled women, as the treatment benefits outweigh the risks.

There is still much confusion about the effect that IBD could have on pregnancy; therefore, it is of paramount importance that women affected with IBD and with the desire to conceive be addressed by proper medical counseling [53]. Compliance with treatment improves in women who receive an appropriate consultation regarding drug therapy before conception and during gestation [69,73]. Thus, gastroenterologists should stimulate discussion about concerns related to IBD and pregnancy, reassuring patients about treatment safety. Overall, the importance of maintaining disease remission should be emphasized for the best pregnancy outcome.

## 9. IBD and Female Hormones

Fluctuation in ovarian hormone levels influences visceral hypersensitivity, GI transit time (via sex hormone receptors), and pain perception (via opioidergic and serotonergic systems) [74,75]. Puberty, pregnancy, and menopause are the three phases in a woman’s life in which sex hormones have a crucial role and influence IBD symptoms and outcomes [3]. Hormones, such as 17-beta estradiol (estrogen), prolactin, and testosterone, are considered directly involved in symptom variation, albeit molecular and cellular mechanisms involved in IBD pathogenesis are still poorly understood. Moreover, the activation of estrogen receptors expressed by epithelial cells contributes to the increase of gut permeability and the activation of humoral and cellular immunity [74].

Notably, the estrogen receptor beta (ERb) seems to have a role of paramount importance in IBD. The ERb is highly expressed in colonic epithelial cells, thereby preserving tight-junction organization, mucosal structure, and barrier function. Interestingly, its expression is markedly reduced in the colonic mucosa of CD/UC patients with active disease [76]. Upon ligand binding, ERb translocates into the nucleus and regulates the transcription of target genes [3]. In an experimental model of CD-like ileitis, Goodman et al. found that ERb protected male but not female mice. Conversely, ERb activation was associated with an anti-inflammatory effect in female but not in male UC models. The molecular mechanisms underlying ERb signaling, and, in general, intestinal inflammation may explain the gender gap observed in the UC incidence, as seen in CD [77].

The different phases of the menstrual period affect GI symptoms cyclically, and menstruation worsens GI symptoms, primarily diarrhea, in IBD women. Indeed, studies investigating the effect of IBD disease activity and medications on GI symptoms during the menstrual cycle found a correlation between disease activity and the worsening of GI symptoms [78]. Consequently, treatment of menstrual disorders with non-steroidal anti-inflammatory drugs (NSAIDs) and oral contraceptive pills (OCP) may influence the IBD course. Women affected by IBD showed a delayed onset of puberty and irregularities in menstrual function (e.g., menstrual abnormalities, oligomenorrhea, and polymenorrhea) [74,79]. Interestingly, alterations in the menstrual cycle can occur a year before IBD diagnosis and are favored by corticosteroids [80]. The mechanisms that evoke menstrual cycle abnormalities are yet to be clarified; possibly, the stress associated with a chronic disease, surgeries, and nutrient malabsorption could play a role. Surprisingly, symptoms improved with the increase in disease duration. Studies on the contribution of menopause and hormone replacement therapy (HRT) on IBD disease activity are few and inconsistent. Some evidence suggested that CD could anticipate menopause [81], while others found no differences between IBD women and healthy controls [82]. Thus, menopause has little or no effect on disease activity and flares.

Research evaluating the association between IBD and HRT found a decrease in the risk of flares during the first two years after menopause, a phenomenon likely promoted by the anti-estrogen action known to exert inflammatory properties [83]. Conversely, a study on American women found a correlation between the use of HRT and the risk of developing UC, but not CD [84]. HRT-UC relation could be allegedly induced by the effect of estrogens on intestinal permeability, immune function, and influence on gut microbiota.

Few studies have also investigated the role of OCP on IBD flare-ups. One study of 152 CD patients reported an increased risk of relapse among CD patients taking OCP [85]. In contrast, another study of 331 women aged 16–50 found no increase in the risk of relapse in patients with CD on OCP treatment [86]. In this subset, it has been speculated that the increased risk of CD in patients may be due to the effect of estrogen on venous hypercoagulability. In addition, estrogen may enhance the development of T helper 1 (Th1) and/or T helper 2 (Th2)-mediated inflammatory diseases. Finally, the modification of the gut microbiome should be addressed. The increased risk of CD in premenopausal women on OCP and the risk of increased UC in postmenopausal women on HRT could explain the differences in the hormonal milieu during each state [87].

## 10. IBD and Menstrual Cycle

Studies investigating the effects of medications used to treat IBD and menstrual function are inconclusive. The use of adalimumab (ADA) has been reported to be effective in restoring menstruation in a 36-year-old female with ankylosing spondylitis and premature ovarian failure [88]. However, the same drug had an adverse effect on menstrual function in a 32-year-old woman with menorrhagia and menstrual pain [89]. The use of anti-TNF-α has been investigated in patients with endometriosis, as this condition is thought to share common features with immune-mediated diseases such as IBD. There is little available data about this possible relationship. However, a trial assessing pain scores in 21 women with endometriosis randomly assigned to either infliximab or placebo found no difference between the two groups [90]. Moreover, the limited evidence between IBD surgery and menstrual function suggests that surgical procedures (i.e., bowel resection and anastomosis, and, respectively, stricturoplasty for CD and IPAA for UC) have a negative impact on menstrual function. A study evaluating menstrual abnormalities in 662 women who underwent surgery for IBD revealed that 60% of women with CD and 53% with UC experienced menstrual cycle abnormalities [79]. 

There is a lack of data on the effect that bowel resection and anastomosis have on the menstrual cycle, and for CD, there is a need for research investigating the role that strictureplasty could exert on menstrual function. Findings on the effect of colectomy on menstrual irregularities in UC patients showed no or minor menstrual irregularities after surgery with resolution over time [64,91]. Moreover, few studies suggested an association between CD and endometriosis in the ileum and colon [92]. A study including 37,661 women hospitalized for endometriosis [93] supported a link between this condition and IBD. The co-existence of endometriosis and IBD could be explained by their similar immunological features and the use of OCP to treat endometriosis.

Furthermore, data on dysmenorrhea and IBD are scarce. A study investigating 44 CD patients and 66 controls concluded that dysmenorrhea together with CD are prognostic factors for global pain severity, and that patients reported higher pain scores during the menstrual cycle vs. controls [94]. The most used therapy to treat dysmenorrhea in IBD patients is nonsteroidal anti-inflammatory drugs (NSAIDs). The use of such drugs has been debated. Indeed, a study reported an increased absolute incidence of CD and UC in women exposed to NSAIDs for at least 15 days/month, suggesting that these drugs could trigger the onset of IBD [95]. However, findings on the effect of NSAIDs on IBD flares are contradictory. One study on 704 subjects found no relation between the use of NSAIDs and IBD relapse, whereas another study investigating 209 patients found a significant association between NSAIDs and early clinical relapse [96]. Overall, these results suggested a cautionary approach when using NSAIDs and avoiding them if possible. Studies assessing the effect of IBD on women’s fertility and pregnancy are summarized in Table 3.

## 11. IBD and Female Psychology

Psychiatric disorders and psychological distress showed a female preponderance in IBD. Self-reported quality of life questionnaires showed lower scores in females than in males [97,98,99]. A Chinese study involving more than 1000 participants of both genders suggests that the impact of CD in females is related to a lower satisfaction level of quality of care (QoC) due to disease symptoms (e.g., pain and discomfort) and depression [100]. Another study aimed at finding gender-specific concerns in 1102 Swiss IBD patients revealed that cancer risk is the primary concern for both genders. Women >40 years old were not worried about their illness but being unemployed increased their concern [101]. Fatigue, a typical symptom of IBD, seems to be more present in women than men, independently from anemia and the state of activity of the disease [102,103,104,105]. Moreover, sexual activity is reduced in women more than men, mainly because of their impaired sexual body image and libido after surgery [106,107]. However, although females appear to be more prone to psychological disorders, they are also interested in receiving information regarding depression from specialists and media [108]. Additionally, when the disease is active, women report more use of emotion-focused and problem-focused coping than men. Such behavior may depend on the traditional role of family caregivers, which is still strongly present in developed countries. In general, society has always invested women with a clear system of expectations that has distorted the real perception of their nature. However, in recent years, scientific research has begun to investigate women with a more rational and objective approach. In this light, there is a need for future studies that will accompany women’s methods of coping with various diseases in a way that “exceeds” or counters the “normative” set of expectations that has been adapted to the male methods of recovery and coping [109,110].

Studies evaluating psychosocial distress, emotional disturbances and impaired QoL in patients with IBD are summarized in Table 4.

## 12. Conclusions

**Figure 2:**
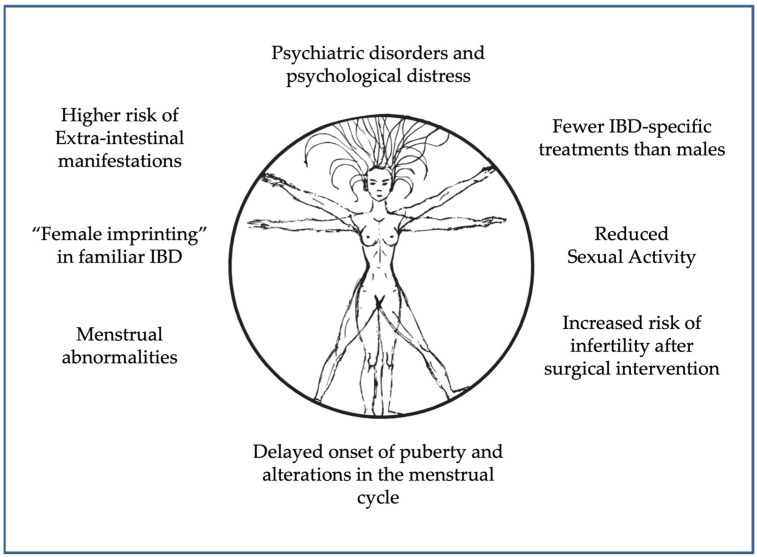
Summary scheme detailing various IBD-related abnormalities known to affect women’s health.

IBDs are chronic disorders with an unpredictable natural history and outcome. Gender-specific differences influence the onset, course, and therapy, as in other immune-mediated disorders, i.e., rheumatoid arthritis, scleroderma, and systemic lupus erythematosus. Moreover, unlike healthy women, females with IBD show an impaired menstrual cycle, reduced libido, and decreased sexual activities. However, although women suffering from IBD present a lower quality of life and a higher rate of psychological disturbances than men, they also show a more proactive attitude to solving their psychological problems and better coping strategies. Awareness of female-related issues in IBD presentation and progression may improve diagnostic and therapeutic strategies to aid women’s health. Gender medicine could be the correct answer to the gender-specific issues arising from IBD patients’ management by improving: (a) IBD diagnosis timing; (b) recognition of gender-specific symptoms; and (c) IBD treatment. Overall, IBD affects many sides of a woman’s health, as summarized in Figure 2.

Unveiling the plethora of complex biological mechanisms promoting the female-specific differences in IBD could foster gender-tailored treatments for IBD.

## Figures and Tables

**Table 1:** Studies assessing the incidence and prevalence of IBD by gender.

Study	Study Population	Outcome
Fujimoto et al., 2007 [5].	844 UC patients.	Male UC patients significantly increased from 1981 to 2000.
Jiang et al., 2006 [6].	63 CD, 389 UC patients.	Male predominance in IBD. Ratio male to female patients = 1.53:1 in UC, 2.32:1 in CD.
Yang et al., 2008 [7].	138 CD, 341 UC patients.	Marked male predominance in CD incidence but not in UC. CD: 102 men, 36 women; UC: 170 men, 171 women.
Gearry et al., 2006 [8].	715 CD, 668 UC patients.	Female predominance in CD incidence but not in UC: CD: 293 men, 422 women; UC: 342 men, 326 women.
Shah et al., 2018 [10].	95,605 CD patients (42,831 males, 52,774 females) 112,004 UC patients (61,672 males, 50,332 females).	No gender difference in UC incidence except for the age group of 5–9 years, until age 45 years; thereafter, men had a significantly higher incidence of UC than women.
Kyle, 1992 [12].	914 CD patients.	Female predominance (63%) in CD incidence.
Latour et al., 1998 [13].	137 CD, 111 UC patients.	Female predominance in CD. CD Female/male ratio: 1.6; UC Female/male ratio: 0.5.
Shivananda et al., 1987 [14].	1040 IBS patients: 210 CD, 257 UC and 573 patients with no disease classification for incomplete data.	Female predominance in CD (male/female ratio = 1:1.33).
Leong et al., 2004 [17].	80 CD patients.	Male predominance in CD (male/female ratio = 2.5:1).
Zelinkova et al., 2012 [19].	608 familial IBD and 415 sporadic IBDs patients.	Females in familial IBD population was significantly higher (61%; female/male ratio = 1.5) compared with sporadic IBD (54%; female/male ratio = 1.2). Significantly higher female to female transmission vs. female to male transmission rate (36 vs. 18). Significantly higher number of mother-to-child transmissions vs. father to child transmissions (55 vs. 32). The female imprinting was specifically related to Crohn’s disease (31 vs. 14 mother vs. father to child transmissions, respectively).
Tedde et al., 2008 [20].	203 UC unrelated patients, 391 controls.	Significant association between mean age at onset of UC and the different IL10-1082 genotypes, observed only in females after gender stratification.
Lin Z. et al., 2010 [21].	106 familial IBDs and 107 sporadic IBDs patients.	Association of IL23R-L310P variant with female UC and female IBD overall, but not with female CD. Association of IL23R-R381Q variant with female CD, but not with female UC, nor with IBD overall. This evidence suggests in females a protective role of IL23R variant R381Q against CD and IBD overall and of the variant L310P against UC.
Jaźwińska-Tarnawska et al., 2015 [22].	61 IBDs patients, 101 controls.	In frequency distributions, significant correlation between gender and C3435T genotype of *ABCB1* gene, both for IBD and CD patients, with 3435CT heterozygote prevailing in IBD and CD males. In odds ratio, significant difference for the 3435CT genotype between control and: IBDs as a whole; IBD males; CD males; and for 3435TT variant in control vs. IBD males. Data suggesting 3435CT genotype as risk factor for IBD and CD in males, while 3435TT genotype in males as protective for IBD.
Friedrichs et al., 2006 [23].	613 CD patients, 749 controls.	Odds ratio for the R30Q variant of *DLG5* gene in CD patients: 2.49 in males; 1.01 in females. R30Q variant constitutes a susceptibility factor for CD in men.
Biank et al., 2007 [24].	281 CD (181 trios) patients, 479 controls in pediatric age.	Significant negative association for R30Q in female children (OR 0.39), but not in male children. DLG5 protective effect in CD for female children.
Vermeire et al., 2001 [26].	79 sibling pairs (68 CD, 11 mixed) were genotyped at 12 microsatellite markers covering the X chromosome. In the second stage, 10 additional markers in the X-pericentromeric region were studied in the families involved in stage 1 together with 62 additional families (52 sibling pairs, 14 s-degree relative pairs).	In the first stage, evidence for linkage was found over a 30-cM pericentromeric region spanning dXs991, dXs990, and dXs8096 (multipoint maximum LOD score in the CD subgroup, 2.5; *p* = 0.0003). The remainder of the X chromosome was excluded (exclusion under LOD-2) for a locus with lambda(s) = 2. Fine mapping in the second stage confirmed linkage and narrowed and shifted the linked region to Xq21.3 around dXs1203 (nonparametric linkage (NPL), 2.90; *p* = 0.0017). The NPL-1 interval around the linkage peak comprises 19.7 cM.
Lee et al., 2017 [27].	1505 IBD patients (922 CD, 583 UC during the discovery phase), and 4041 controls. Additional 1989 IBD patients (993 CD, 996 UC) and 3491 controls.	Significant association of a previously reported inflammatory bowel disease susceptibility locus at chromosome Xq26.3 (CD40LG-ARHGEF6; odds ratio, 1.22; 95% confidence interval, 1.16–1.28; combined *p* = 3.79 × 10^−15^). This locus accounted for 0.18% and 0.12% of genetic variance in CD and UC, respectively, and increased the total autosomal chromosome genetic variance from 6.65% to 6.83% and from 5.47% to 5.59% for CD and UC risk, respectively, in the Korean population. Sex-stratified analyses did not reveal sex-related differences in effect sizes.
Kudelka et al., 2018 [28].	Mice: Cosmcf/y (KO), Vil-Cre+; Cosmcf/+ (mosaic), and Vil-Cre+ (WT) controls.	Cosmc regulates host genes, bacterial ligands, and nutrient availability to control microbiota biogeography. Loss of one Cosmc allele in males (IEC-Cosmc-/y) resulted in a compromised mucus layer, spontaneous microbe-dependent inflammation, and enhanced experimental colitis; however, females with loss of one allele and mosaic deletion of Cosmc in 50% of crypts (IEC-Cosmc+/−) were protected from spontaneous inflammation and partially protected from experimental colitis, likely due to lateral migration of normal mucin glycocalyx from WT cells over KO crypts.

**Table 2:** IBD Clinical features and management.

Study	Study Population	Outcome
Wagtmans et al., 2001 [11].	541 CD patients (266 males, 275 females).	No difference in mean lag time between onset of symptoms and diagnosis, and no differences in presenting symptoms and initial localization of lesions. Similar percentage of patients who underwent an abdominal operation (81% vs. 77%). No difference in mean lag-time between onset of symptoms and first bowel resection. Lag-time between bowel resection and recurrence of disease shorter in women than in men (4.8 yr vs. 6.5 yr), ileocecal resections more frequent in female than male patients (44% and 32%, respectively). Female patients have significantly more often relatives in the first or second degree affected by CD than male (15% vs. 8.3%).
Severs et al., 2018 [18].	Dutch IBD Biobank study: 2118 CD and 1269 UC patients. COIN study: 1139 CD and 1213 UC patients.	Early onset CD (<16 years) more frequent in males than in females (20% vs. 12%). Male CDs have more often ileal disease (28% vs. 20%) and underwent more often small bowel and ileocecal resection. Male CDs used prednisone more often and suffered more often from osteopenia. IBD-specific healthcare costs did not differ between male and female IBD patients. Extraintestinal manifestations more frequent in female IBD patients than male.
Mazor et al., 2011 [33].	146 patients with CD (76 females, 70 males) treated during a 10-year period.	The only independent risk factors associated with developing a complication were smoking and male gender. There was no association between developing complications and the presence of selected SNPs (*p* = 0.07 for tyrosine residue on both alleles in NCF4 SNP rs4821544 and *p* = 0.06 for a guanine residue on both alleles in ATG16L SNP rs2241880). Multivariate analysis using a backwards logistic regression model left only male gender as an independent statistically significant association with complicated disease (OR 2.6017, 95% CI: 1.17 to 5.75). The median time to developing a complication was 4 years, and the most common complication was the need for surgical intervention (54%).
Blumenstein et al., 2011 [34].	986 patients with IBD (515 CD, 471 UC—537 females, 449 males).	Extended disease duration in women, no significant gender-related differences in demographic and clinical characteristics observed. Men showed a significantly higher remission rate than women (*p* = 0.025), while women received significantly less immunosuppressive medication compared to men (*p* = 0.011). Treatment with immunosuppressants was not different in women with child-bearing potential compared to menopausal women.
Bokemeyer et al., 2013 [35].	1032 patients with IBD (511 CD, 519 UC, 2 IBD-U).	About one third of the IBD patients were not in clinical remission (CDAI ≥ 150/CAI > 4) (CD: 45%; UC: 27%), although high rates of immunosuppressive drugs (CD: 47%; UC 26%) were administered. This study shows a large burden of active disease associated with an unexpectedly high (co)morbidity and high psychosocial impairments, indicating a reduced health state in IBD patients.
Greuter et al., 2018 [36].	1638 CD patients (107 presented with upper GI tract involvement at the time of diagnosis, 214 at any time).	In a multivariate logistic regression model, male sex, and diagnosis between 2009 and 2016 (versus before 1995) were independent predictors for presence of upper GI tract involvement at CD diagnosis (odds ratio [OR] 1.600, *p* = 0.021 and OR 2.686, *p* < 0.001, respectively), whereas adult age was a negative predictor (OR 0.388, *p* = 0.001). Patients with upper GI tract involvement showed a disease course similar to control patients (hazard ratio [HR] for any complications 0.887, (95% confidence interval [CI] 0.409–1.920), and a trend towards occurrence of fewer intestinal fistulas (log-rank test *p* = 0.054).
Jussila et al., 2014 [38].	21,964 patients with IBD (5315 CD, 16,649 UC).	Overall mortality was increased among patients with CD (standardized mortality ratio (SMR) 1.33, 95% confidence interval 1.21–1.46) and UC (1.10, 1.05–1.15). SMR was significantly increased for gastrointestinal causes in CD (6.53, 4.91–8.52) and UC (2.81, 2.32–3.34). Patients with UC were found also to have increased SMR from pulmonary (1.24, 1.02–1.46) and cardiovascular disease (1.14, 1.06–1.22) and cancers of the colon (1.90, 1.38–2.55), rectum (1.79, 1.14–2.69) and biliary tract (5.65, 3.54–8.54), whereas SMR from alcohol-related deaths was decreased (0.54, 0.39–0.71). Patients with CD had a significantly increased SMR for pulmonary diseases (2.01, 1.39–2.80), infections (4.27, 2.13–7.63) and cancers of the biliary tract (4.51, 1.23–11.5) and lymphoid and hematopoietic tissue (2.95, 1.85–4.45).
Peyrin-Biroulet et al., 2013 [39].	310 patients with CD (154 females, 156 males).	The cumulative probability of major abdominal surgery was 38, 48, and 58% at 5, 10, and 20 years after diagnosis, respectively. Baseline factors significantly associated with time to major abdominal surgery were: ileocolonic (hazards ratios (HRs) 3.3), small bowel (HR, 3.4), and upper gastrointestinal (HR, 4.0) extent, relative to colonic alone; current cigarette smoking (HR, 1.7), male gender (HR, 1.6), penetrating disease behavior (HR, 2.7), and early corticosteroid use (HR = 1.6). Major abdominal surgery rates remained stable, with 5-year cumulative probabilities in 1970–1974 and 2000–2004 of 37.5 and 35.1%, respectively. The cumulative probability of major abdominal surgery in this population-based cohort of Crohn’s disease approached 60% after 20 years of disease, and many patients required second or third surgeries. Non-colonic disease extent, current smoking, male gender, penetrating disease behavior, and early steroid use were significantly associated with major abdominal surgery.
Walldorf et al., 2013 [40].	293 patients with IBD (195 CD, 98 UC—110 males, 183 females).	DEXA-scan was performed in 174 patients (59 males, 115 females). Bone mineral density (BMD) was impaired in 38.5% of these patients. Male patients were diagnosed more often with osteopenia or osteoporosis than females (55.9% vs. 29.6%, *p* = 0.03) and had a risk of bone disease comparable to postmenopausal women. Additionally, duration of corticosteroid treatment and IBD were identified as risk factors for osteoporosis. Follow up DEXA-scan demonstrated an overall deterioration of BMD in patients with normal baseline results.
Sigurdsson et al., 2022 [42].	49 young adult male patients with childhood-onset IBD and 245 matched controls.	The group of young adult patients had, in comparison with the controls, significantly smaller median cortical area (126.1 mm^2^ vs 151.1 mm^2^, *p* < 0.001), lower median total vBMD (296.7 mg/cm^3^ vs. 336.7 mg/cm^3^, *p* < 0.001), and lower median cortical vBMD (854.4 mg/cm^3^ vs. 878.5 mg/cm^3^, *p* < 0.001). Furthermore, the patients compared with the controls had lower median trabecular volume fraction (16.8% vs. 18.2%, *p* < 0.001) and thinner median trabeculae (0.084 mm vs. 0.089 mm, *p* < 0.001). The differences between the patients with IBD and controls persisted in multivariable analyses that included adjustments for SMI and physical exercise.
Heath et al., 2022 [50].	1015 patients; 656 CD (59.0% women) and 359 UC (47.9% women).	Women were more likely prescribed budesonide than men (23.6% vs. 13.4%; *p* < 0.0001), more men were exposed to prednisone for IBD management (73.5% vs. 67.4%; *p* = 0.04). Immunomodulator use was higher in men with CD versus women (86.6% vs. 78.3%; *p* = 0.008) and of those exposed, women more commonly experienced ADRs (29.5% vs. 21.2%; *p* = 0.01). Though no sex-related difference was identified, age was a predictor of biologic exposure in women with CD and men with UC, with those > 55 being less likely to receive biologics.

**Table 3:** Studies evaluating the effect of IBD on women’s fertility and pregnancy.

Study	Study Population	Outcome
Baird et al., 1990 [55].	261 women with IBD, (177 CD, 84 UC) matched controls.	No evidence of increased risk of pregnancy loss. The risk of preterm birth was significantly elevated for patients with CD (odds ratio, 3.1; 95% confidence interval, 1.8–5.4) and for those with UC (odds ratio, 2.7; 95% confidence interval, 1.8–5.4).
Hudson et al., 1997 [56].	409 women with IBD (177 CD, 232 UC).	Women with UC and CD had normal fertility when compared with the general population. However, unresolved infertility problems were more frequent in women who had undergone surgery for inflammatory bowel disease compared with those who had not (12% vs. 5% for Crohn’s disease; 25% vs. 7% for ulcerative colitis). Disease relapse rates did not increase in pregnancy.
Khosla et al., 1984 [57].	112 married women with CD.	Infertility rate (12%) similar to the general population. Patients in remission during conception had a normal pregnancy. In the majority CD remained quiescent.
Marri et al., 2007 [58].	169 females aged 15–44 years with IBD (110 CD, 59 UC).	The rates of no voluntary childlessness in IBD were similar to the general population. Women with IBD also had fewer children than their national counterparts.
Fréour et al., 2012 [59].	Serum levels of AMH were measured (50 women with CD in remission, 163 controls).	Women with CD do not have severe ovarian reserve alterations compared to a control population. Age ≥ 30 years and a colonic location of the disease could be associated with an accelerated loss of follicles.
Ban et al., 2015 [60].	9639 women with IBD (4475 CD, 4354 UC), and 2,131,864 controls.	Women with CD have marginally lower fertility rates. These rates decreased following flares and surgical interventions. Fertility rates returned almost to normal when women were not prescribed contraception but the reduction following surgical intervention remained.
Şenateş et al., 2013 [61].	Serum AMH levels measured in 35 women with CD and 35 age-matched controls.	AMH levels in CD patients (1.02 ± 0.72) were significantly lower compared to the controls (1.89 ± 1.80) (*p* = 0.009). Serum AMH levels in CD patients with active disease (0.33 ± 0.25) were significantly lower compared to CD patients who were in remission (1.53 ± 0.49) (*p* = 0.001). In CD patients, a negative correlation between CD disease activity and serum AMH levels was found (r = −0.718, *p* < 0.001).
Lee et al., 2020 [62].	2058 women with IBD (589 CD, 1469 UC) and 20,580 age-matched controls.	With the exception of moderate to severe disease, the incidences of adverse pregnancy outcomes in women with IBD are similar to that of the general population.
Oresland, 1994 [64].	21 women with UC operated with endoanal mucosectomy and a handsewn ileal pouch-anal anastomosis.	Gross abnormalities were seen on hysterosalpingography but were of a magnitude no greater than that after the conventional proctocolectomy. 2/3 of the women who attempted to become pregnant, failed in five years of fu, indicating a high incidence of infertility.
Wikland, 1990 [66].	71 women who had a proctocolectomy (30 CD, 41 UC).	Fertility was significantly reduced after surgery since only 37% (10/27) of the women who attempted to become pregnant succeeded within 5 years follow-up. Conventional proctocolectomy in women will result in distressing vaginal discharge, and dyspareunia in a considerable proportion of the patients. The operation seems to decrease their chances of becoming pregnant.
Mountifield et al., 2009 [67].	255 patients (72 males, 183 females- 127 CD, 85 UC, 5 IBD-U).	The average fertility rate was no different between women with CD and UC (1.0 and 1.2 births/woman, respectively; *p* = 0.553), compared with 1.81 for all Australian women. Although 42.7% of IBD patients reported a fear of infertility, patients only sought medical fertility advice at the same rate as the general population. Fear of infertility was most evident in women, those with CD, and those reporting previous surgery. Specific patient concerns, which appear to have decreased patients’ family size, included IBD heritability, the risk of congenital abnormalities, and medication teratogenicity.
Bortoli et al., 2011 [70].	332 pregnant women with IBD (145 CD, 187 UC) and 332 controls.	No statistically significant differences in frequency of abortions, preterm deliveries, caesarean sections, congenital abnormalities, and birth weight were observed comparing CD and UC women with their non-IBD controls.
Van der Giessen et al., 2011 [72].	IBD patient-derived inflammatory organoid models and 2D cell lines models.	Progesterone and estrogen improved wound healing and epithelial barrier function in intestinal epithelial cells via upregulation of tight junction proteins. Furthermore, these sex hormones significantly reduced ER stress and reduce pro-inflammatory cytokine production in intestinal epithelial models. The study shows that estrogen and progesterone alleviate ER stress, decrease pro-inflammatory cytokine production, stimulate wound healing, and increase barrier function of epithelial cells. Pregnancy hormones can have beneficial effects on disease activity by positively modulating the intestinal epithelial lining.
Julsgaard et al., 2011 [73].	115 women with UC who gave birth.	Adherence to the therapy was high despite fear of a negative effect on fertility or the fetus. Counseling predicted higher adherence. This may be important because our study suggests an increase in UC activity during pregnancy.
Pierdominici et al., 2015 [76].	Intracellular expression of ERα and ERβ in peripheral blood T lymphocytes from 48 patients with IBD (26 CD, 22 UC) and 29 healthy controls.	Significant reduction (*p* < 0.05) in estrogen receptor (ER) β expression in peripheral blood T lymphocytes from CD/UC patients with active disease (*n* = 27) as compared to those in remission (*n* = 21) and healthy controls (*n* = 29).
Goodman et al., 2014 [77].	SAMP1/YitFc (SAMP) mice—model of chronic intestinal inflammation similar to human CD.	Conventional T cells (Tconv) and Tregs responded differentially to estrogen signaling, leading to distinct immunoprotective effects mediated by distinct estrogen receptor (ER) isoforms. These mechanisms were impaired in T cells from SAMP-F mice. Thus, hormone signaling influences the expansion and function of GALT Tregs in an ER-dependent manner and contributes to gender-based differences in experimental CD.
Webe et al., 1995 [79].	662 women with IBD (360 CD, 251 UC, 51 IBD-U).	60% of women with CD and 53% with UC experienced menstrual cycle abnormalities. Gynecologic conditions are common in women with inflammatory bowel disease, including menstrual abnormalities, vaginal discharge, infertility, and gynecologic surgery.
Saha et al., 2014 [80].	121 women with IBD.	25% of patients experienced a change in cycle interval in the year before IBD diagnosis and 21% experienced a change in the duration of flow. Among women with dysmenorrhea, 40% experienced a change in the intensity of their menstrual pain, 31% experienced a change in its duration. Overall cycle regularity increased over time. Changes in menstrual function occur frequently in the year before IBD diagnosis.
Lichtarowicz et al., 1989 [81].	146 women with CD.	A logistic analysis showed that 50% of women with CD had the menopause at 47.6 years compared with 49.6 years in a group of healthy women from the same area. The two groups had similar smoking habits. Premature menopause seems associated with CD.
Khalili et al., 2012 [84].	108,844 postmenopausal women without a prior history of CD or UC.	Postmenopausal hormone therapy was associated with an increased risk of UC but not CD. These findings indicate that pathways related to estrogens might mediate the pathogenesis of UC. HR for UC was 1.71 (95% CI, 1.07–2.74) among women who currently used hormones and 1.65 (95% CI, 1.03–2.66) among past users.
Timmer et al., 1998 [85].	152 patients with CD, 88 women.	Unfavorable outcomes for women (*p* = 0.05), current smokers (*p* = 0.005), and use of oral contraceptives (*p* = 0.001) for what concerns relapses in patients with CD.
Cosnes et al., 1999 [86].	331 women with CD.	Unlike smoking, oral contraceptives have no effect on CD activity.
Khalili et al., 2013 [87].	117,375 women enrolled since 1976 in the Nurses Health Study I (NHS I) and 115,077 women enrolled since 1989 in the Nurses’ Health Study II (NHS II) with no prior history of UC or CD.	Oral contraceptive use was associated with risk of CD.
Saha et al., 2013 [94].	110 women 40% had dysmenorrheal (54 CD, 66 controls).	Dysmenorrhea is common in women with CD and has an additive effect on overall pain severity. Menstrual distress is positively correlated with CD activity scores and associated with lower HRQOL by some measures.
Ananthakrishnan et al., 2012 [95].	76,795 women who provided data about aspirin and NSAID use.	123 incident cases of CD and 117 cases of UC occurred over 18 years and 1,295,317 person-years of follow-up. Frequent use of NSAIDs but not aspirin seemed to be associated with increased absolute incidence of CD and UC.
Takeuchi et al., 2006 [96].	209 patients with IBD.	Nonselective NSAIDs were associated with a 17–28% relapse rate within 9 days of ingestion. No patient had an early relapse on acetaminophen, nimesulide, or aspirin, whereas those on naproxen and nabumetone (20%) experienced relapse. These clinical relapses were associated with escalating intestinal inflammatory activity.

**Table 4:** Studies evaluating psychosocial distress, emotional disturbances and impaired QoL in patients with IBD.

Study	Study Population	Outcome
Nurmi et al., 2013 [97].	556 patients with IBD (292 females, 264 males).	Women had seen doctors more often than men (*p* < 0.001). Women were absent from work more frequently than men (*p* = 0.01). The amount of physician visits, work absenteeism, and a higher amount of undergone procedures were related to impaired HRQoL (*p* < 0.001 on all accounts).
Graff et al., 2006 [98].	388 patients with IBD.	Multivariate regression showed that those with active disease had higher levels of distress, health anxiety, and perceived stress, lower social support, well-being and mastery, and poorer disease-specific QOL, relative to those with inactive disease. Participants with either active or inactive disease had suboptimal general QOL.
Hauser et al., 2011 [99].	112 IBD patients (51 CD, 61 UC—50 females, 62 males).	Women have expressed significantly lower level of the general HRQoL and more emotional disturbances connected with their disease as well as more frequent bowel symptoms compared with men.
Yan et al., 2020 [100].	891 IBD patients (522 CD, 363 UC, 6 IBD-U—362 females, 529 males).	Female patients showed a higher tendency to feel that the quality of communication with specialists (*p* = 0.037) and quality of IBD care (*p* = 0.019) was less satisfactory than male patients. Female patients with IBD show a larger number of intense concerns, a greater level of psychological disturbance, a higher symptom load, and a poorer QoL than men, resulting in reduced satisfaction ratings.
Pittet et al., 2017 [101].	1102 IBD patients (596 CD, 475 UC, 31 IBD-U—598 females, 504 males).	Women had significantly higher overall levels of concern than did men (sum score: 47.5 vs. 42.8, respectively, *p* < 0.001). Women at home or unemployed had higher concerns about disease-related constraints and uncertainty (*p* = 0.004). Patients seem to have important gender-specific concerns related to their illness.
Saraiva et al., 2019 [102].	105 IBD patients (60 CD, 45 UC—60 females, 45 males).	Female gender and active CD were significantly associated with a severe level of fatigue (*p* = 0.05 and *p* = 0.04).
Bager et al., 2012 [103].	425 IBD patients (251 CD, 174 UC).	Female IBD patients tend to experience more fatigue than males. When comparing the IBD patients with disease activity to the IBD patients in remission, all dimensions of fatigue were statistically significant (*p* <0.05). Fatigue in IBD is common regardless of anaemia or iron deficiency. Fatigue in IBD is most marked for patients <60 years of age. Fatigue is expressed differently between groups.
Le Berre et al., 2019 [104].	1410 IBD patients (875 CD, 496 UC, 39 IBD-U).	Among the disabling symptoms at work, fatigue was the most frequent (41%) followed by diarrhea (25%) and fecal incontinence (18%). IBD has a strong negative impact on working life. While work satisfaction remains high, IBD affects career plans.

## Data Availability

Not applicable.
